# Reciprocal cognitive and emotional interaction in STEMM university learning and teaching

**DOI:** 10.1038/s41598-024-72656-w

**Published:** 2024-09-17

**Authors:** Kate Ippolito, Martyn Kingsbury

**Affiliations:** Imperial College London, London, UK

**Keywords:** Human behaviour, Emotion

## Abstract

University learning and teaching involves contrasting and interacting emotional experiences. Even in disciplines considered as objective as Science and Engineering, emotion plays a significant role in catalysing and sustaining learning. Although emotions are individually felt, they are socially constructed between people. This is especially relevant in group-based learning evident in much contemporary higher education. This paper applies the concept of emotion socialization to illustrate how groups of students and teachers cope and succeed in cognitively and emotionally challenging learning settings. The study is based on qualitative data collected across six STEMM university departments, from 280 students via in-situ questionnaires and from 20 teachers via group and follow-up interviews. Two key findings offer insight into processes of reciprocal influence on cognition and emotion. The first relates to ways in which students’ and teachers’ differing knowledge-related goals and relationships with knowledge influenced cognition and emotion, uncovering previously unacknowledged connections. The second relates to how students’ comparisons of progress towards academic goals with peers contributed considerably to their emotional experiences in cognitively and emotionally helpful and unhelpful ways. Practical implications are presented, including possibilities for capitalising on existing emotion socialization processes and enhancing how they influence cognition and emotional well-being.

## Introduction

University learning and teaching involves contrasting and interacting emotional experiences. Students may experience confusion over challenging concepts, joy at grasping them, anxiety about assessment and pride in group projects. Similarly, teachers can feel excitement or apprehension about using new techniques and frustration at perceived lack of student engagement. Although emotions are individually felt, they are socially constructed between people^1^. Emotions and the appraisals that influence them are very often related to other people and regulated in association with others^2^. This is especially relevant in educational contexts, and more so in the interactive, group-based learning often practised in contemporary higher education^3^. We argue that, far from being something to avoid or ignore, emotions provide important social^4^ and educational information within our classrooms that supports academic coping and success. Despite this, there is little systematic thinking about how students’ and teachers’ emotions influence each other and the impact this has on the learning, and social and emotional well-being of classroom-based groups. This paper presents research on how student and teacher cognition and emotion continually reciprocally interact to enable the group to function in challenging academic contexts.

### Emotion in learning

Over the last 25 years, thinking has progressed from a perception that emotion obstructs learning, to understanding that it is ‘neurobiologically impossible’ to engage in cognitive processes such as paying attention, remembering material, problem-solving and decision-making without emotion^5^. Even in scientific disciplines, traditionally considered objective, rational, dispassionate, with little place for emotion, the catalyst to start and motivation to persist in learning and scientific enquiry is emotion; curiosity, confusion and anticipated joy. Advances in neuroscience reveal that ongoing brain development and associated learning are dependent on integrated socioemotional and cognitive functioning across three key brain networks (executive control network, default mode network and salience network)^6^. Emotional stress in response to exams, coursework deadlines and group conflict have contrasting outcomes across cognitive processes including memory formation, retention, retrieval and updating, and between learners^7^. From a psychological perspective, not only is emotion an unavoidable feature of stepping into any learning setting, it is also essential for academic accomplishment and personal development^8^. This is especially true of the transformative learning expected of higher education^9^. If learners never feel the discomfort of uncertainty or satisfaction of achievement, how can they be motivated to change themselves and face related challenges in more developed ways in future? Furthermore, if we accept that failure is both an inevitability and strength of scientific endeavour^10^, we need to equip learners to accept and harness the discomfort and difficult emotions that failure may provoke. Understanding the role of emotion could enable us to capitalise on those that support scientific and technical learning and sustain motivation towards careers in scientific fields^11^.

In Barrett’s^1^ ‘Theory of Constructed Emotion’, she argues that emotion is constructed by individuals based on their physiological sensations, social context, previous experience and their goals, and refined through continual predication and feedback loops. We not only actively construct our own emotional experiences, we also contribute to the constructions of those we interact with^1^. Pekrun’s ‘Control Value Theory’^12^ focuses on how academic achievement emotions, such as enjoyment, pride, shame and anxiety, result from our appraisals of our perceived control and the value we place on achieving our goal. For example, a learner’s appraisal and resulting emotion may relate to their perception of control over an assessment task, or the personal value they perceive in the topic, including how relevant it is to achieving their goals. This theoretical framework for thinking of emotional experience as closely linked to individual and collective goals is helpful in a goal-orientated place like university. It is also timely to critically consider the complex and contradictory roles of emotion in learning, as higher education institutions internationally grapple with the tensions involved in addressing rising student mental health concerns^13^, whilst creating challenging and meaningful learning opportunities.

### Emotion socialization in a university context

Emotion socialization^14^ is a dynamic process in which ‘socializing agents’^15^ facilitate development of emotional competence, including ‘community-relevant’ ways of understanding, expressing and regulating emotions^16^. They do this through the ways that they react to others’ emotional expressions, through talking about emotional responses and through their own emotional displays^16^. By applying the concept of emotion socialization, this study illustrates how, as socializing agents, university students and teachers learn together to work with emotion in purposefully challenging learning and teaching settings.

Just as students do not arrive at university study with all the appropriate disciplinary knowledge, they also do not necessarily possess the emotional competence to cope and thrive at university. They develop community-relevant emotional competence through experiencing, thinking and being in a particular social context. Not only does this process involve cognition and metacognition related to regulation of emotion^17^, it also contributes to students’ ability to develop increasingly advanced ways of acquiring scientific and technical knowledge and understanding. Likewise, as teachers’ contexts and goals change they are continually developing their ability to respond to their own and others’ emotions^18^. Through this process they can develop shared goals, begin to identify as a group and, as individuals identifying as members of that group, experience intergroup emotions^19^.

Despite its exploration in other educational settings, emotion socialization is under-researched in the university context, but we argue it is highly relevant, for a number of reasons:

1. The active and collaborative learning approaches increasingly characterising higher education are designed to actively engage learners both cognitively and emotionally^20^ and this has implications for both learners and teachers^21^.
2. Many students relocate away from primary socializers such as parents, existing peers and schoolteachers for the duration of their studies.
3. University environments are very international, bringing together individuals with diverse and unfamiliar culturally-specific emotional display rules^22^.
4. Due to the relationship between emotion regulation and well-being^23^, it is beneficial that students learn to regulate emotions they experience in learning as part of that learning.
5. Many students are between adolescent and early adult years, a developmental stage often characterised by high emotional reactivity^24^ and greater awareness of others’ emotions^25^.
6. The importance of emotion socialization for developing professional competency to handle complexity is recognised^26^.

## Methods

### Study design

This qualitative study was set in a research-intensive university specialising in Science, Technology, Engineering, Maths and Medicine (STEMM) in the UK. Six case studies were purposefully selected from six departments across the Faculty of Natural Sciences (FoNS), Faculty of Engineering (FoE) and Faculty of Medicine (FoM). Each focussed on a module that involved participants in active, collaborative learning, as opposed to a more traditional, didactic approach. A recent institution-wide curriculum change process^27^ resulted in increased collaborative learning approaches that are new and challenging for students and teachers, due to their unfamiliarity and associated socioemotional interaction^28^. The learning activities under focus in this study are detailed in Table 1.

**Table 1:** Summaries of learning and teaching contexts from six departmental case studies.

Discipline	Year group	Teaching and learning approach
Chemistry FoNS	Year 1	Students work in wet lab-based small groups to develop practical chemistry skills, including chemical synthesis techniques, computer modelling, data analysis and planning their own experiments. This is prefaced by ‘Chemical Kitchen’ that introduces students to the creative mindset and fundamental lab skills through cooking^29^
Electrical and Electronic Engineering FoE	Year 1	Group-based engineering design project during final summer term, involving students building robotic moon rovers and culminating in a demonstration
Life Sciences FoNS	Year 2	Students work in consistent small groups, using a Team-based Learning (TBL) approach to learn statistics and programming. This involves pre-work, individual tests, team tests with immediate feedback and application activities^28^
Mechanical Engineering FoE	Year 2	Intensive week-long, team-based project based on an engineering design consultancy firm simulation. Activities involve students experiencing the whole design process, applying fundamental knowledge and facing uncertainty created by an authentic brief
Medicine FoM	Year 1	Students work in consistent small groups using a combined Case-based Learning (CBL) and Team-based Learning (TBL) approach. This involves pre-work, individual tests, team tests with immediate feedback and application activities. Cases are designed to develop clinical reasoning skills, underpinned by scientific knowledge and evidence-based research^30^
Physics FoNS	Year 1	Students work in consistent small groups in bi-weekly seminars, linked to a lecture series, to solve problems designed to require collaborative thinking. This session specifically involved students developing concept maps to aid collaborative recall and connect physics concepts

### Data collection

Data was collected between April 2020 and February 2023 using the following three step process. First, 90-min online focus group interviews were conducted with six trios of volunteering teachers (5 female and 13 male), to explore their awareness of the role of emotion in their module’s learning and teaching and their approaches to working with emotion (see supplement for Teacher focus group interview schedule). Second, at the end of a teaching session, chosen by each module lead, students from these six modules were invited to complete a voluntary free-text, in-situ anonymous questionnaire administered in MSForms using a QR code. This asked students questions about how the session or project made them feel, why they felt this way, how their teachers and peers contributed to this feeling and how they thought teachers and peers felt (see supplement for Student questionnaire). Students were advised that this piece of research was independent to their learning but that completing the survey could be an educationally valuable reflective activity for them. We explained that anonymous responses would be shared with their teachers to inform future development. Students were shown the survey questions in advance of volunteering and told that submission implied consent. Across the six case studies, 280 students completed the questionnaire. Third, anonymous student data corresponding to each module was presented back to members of the module’s teaching team for consideration and a 60-min interview was held to explore meaning in the student data and identify areas for further consideration. Imperial College Education Ethics Review Process (EERP) committee approved the research (EERP1920-066), all research was performed in accordance with British Educational Research Association guidelines and informed consent was obtained from all participants.

### Data analysis

Interviews with teachers were recorded, transcribed verbatim and coded in NVivo14 and student questionnaire data was collated and coded in Excel. Data was analysed using reflexive thematic analysis^31^, that involved developing codes to capture units of meaning across the data sets, guided by our research questions: What are the reciprocal emotional interactions occurring in STEMM university classrooms? What are the roles of both students and teachers? As the perception that STEMM students and teachers reciprocally influence each other emotionally was based on the researchers’ experience and was not empirically informed, an inductive, or data-driven, orientation, as opposed to a theory-driven approach, was adopted^32^. The coding was developed from students’ and teachers’ responses that referred to the various ways in which students and teachers influenced each other’s emotion and thinking in these six learning settings. Data was coded by one author (KI), and data and codes discussed with MK to develop appropriate themes. In line with reflexive thematic analysis, the goal was not interrater reliability or coding to consensus^31^. From this we developed sub-themes and themes, based on our interpretations of patterns in meaning across the data sets, in association with our knowledge of the context (see supplement for Coding guide). Student quotes are reproduced as typed, to retain authenticity. Data analysis was an iterative process, including a social constructionist element, when meaning of student data was constructed between researcher and participant teachers in follow-up interviews^32^. This methodological approach is well-aligned with our perspective of emotion as being socially constructed. Validity was increased by the researchers’ in-depth awareness of these teaching and learning contexts through their role as educational developers, and the recursive nature of data collection that related and triangulated data sets at every stage^33^. We identified two key themes that illuminate processes of reciprocal influence on cognition and emotion in STEMM university classrooms and the roles of students and teachers. These are: Relationships with knowledge and Cognition and emotion-based comparison**.**

## Results

### Relationships with knowledge

#### Developing cognitive goals

The data indicated that students’ strongest and most varied emotions were constructed in relation to grappling with unfamiliar, voluminous, complex, and sometimes ambiguous knowledge. For example, the responses of 65 Life Sciences students learning about statistics and programming reported a varied emotional continuum of experiences that represent three broad groups of responses. One group described feelings of ‘getting lost’ and ‘falling behind’, based on their perceived inability to readily grasp, easily recall or competently apply scientific and technical concepts, as represented by Student117:

“It made me feel really stupid if I have to be honest – I feel so lost all the time and there are so many words and rules to remember. The teaching makes sense in class but as soon as I step out I am completely lost again. I want to say I’m motivated but seeing everyone miles ahead of me in understanding is really demotivating” (Student117, FoNS).

Representative of the second group, Student77’s positive emotional experience was based on their sense of relative progress and cognitive development: *“I feel decent after this session[…] I have for once fully understood the content, and mostly caught up with the speed of my teammates” (Student77, FoNS).*

The third group’s responses reflected greater awareness that learning is characterised by mixed pleasant and unpleasant valance, including some discomfort. In the quote from particularly insightful Student118 they refer to a cycle of emotion and its value for self-development:

“It made me feel excited to learn, a bit sad for how slow I interpret the content and happy when I solve a question on my own[…]It is kind of a circle. You get discouraged a bit in the beginning especially when you realize how stupid you are. But when you get it done, you will regain and reconstruct your confidence, an even stronger one than before” (Student118, FoNS).

The insights data gave into the diverse range of student emotions and their knowledge-related attributions, even in the same setting, suggested a variety of cognitive goals. For Student117, a goal seemed to be to remember ‘words and rules’, Student77 aimed to fully understand the content and Student118 wanted to problem solve independently, and recognised the link between academic goal achievement and an affective dimension, such as resilient self-efficacy building^34^. We suggest that students constructed and made meaning of their emotions partly in relation to adapting to new, university appropriate, cognitive goals. Some students’ responses implied a recognition that knowledge they were expected to engage cognitively with was now more extensive and less defined, and that there was constructive potential in thinking together with peers, but that this presented emotional challenges, as Student184’s quote illustrates:

“Eager to learn more as I see the areas I don’t know in the depth I should. Overwhelmed as we don’t have time to complete to a high enough level the work that we are set [and] because I don’t know perfectly all the content that I have been taught whilst at imperial. [The teacher] increases the unknown factor by not specifying most things. It’s nice when they [peers] have knowledge in an area you don’t so that we complement each other. But sometimes they slow down the process due to them seeing things differently” (Student184, FoE).

This transition to developing new cognitive goals seemed to involve formulation of contradictory goals and associated mixed emotions regarding knowledge-related standards and expectations, perceived academic ability and knowledge construction with peers. Across the whole dataset, students who reported valuing their interactions with peers as supportive of their cognitive development reported more positive emotional experiences. Student66’s quote illustrates the valuable interplay between cognitive and emotional support that some students reported exchanging, including through gaining awareness of other’s cognitive and emotional challenges:

“It made me feel a bit scared before attending class but during the session I felt reassured[...] My peers also contributed to this feeling to a large extent as talking with them and working through the problems together made me feel united as they also did not understand some of the content initially” (Student66, FoNS).

#### STEMM teachers not knowing everything

Data suggested that teachers also held differing and contradictory cognitive goals. Teacher12’s quote illustrates the emotional discomfort created by misalignment between a teacher’s goal to meet students’ knowledge-related needs and expectations regarding appearing knowledgeable, and their own epistemic belief that, at this level, no one knows everything:

“Personally, I really, really struggle with those moments when the students ask me something that I can’t answer. I really, really hate that[…]obviously, I cannot know everything. But still, I just can’t deal with the feeling” (Teacher12, FoE).

Whereas Teacher10’s quote below represents a completely contrasting emotional response and approach. It also illustrates how teacher participants demonstrated complex cognitive processes in relation to student emotion, including judging how to apply emotional knowledge in their teaching, in context appropriate ways:

“I don’t enjoy maths. And actually, that’s the only approach that I’ve kind of felt confident taking with this, is to be completely honest and say, ‘I'm not a mathematician[…]I'm very much in the same boat as you. And we’ll work through this stuff together.’ And I think they, I think they appreciate it” (Teacher10, FoNS).

Although Teacher10 is highly numerate, they explained that in their department even senior faculty are open about their academic specialism not being maths-based. This approach may facilitate helpful emotion socialization around mathematical knowing and seemed to contribute to Teacher10’s willingness to explicitly model to students how they cope with maths-related discomfort. Teacher10 also acknowledged that realising that academics do not know everything within their chosen discipline must be disorientating for students.

Although many teachers referred to modelling making mistakes and normalising not knowing everything, this is not emotionally neutral in academically demanding STEMM contexts and can create a cognitive and emotional load, as Teacher9 explains:

“I give live demonstrations myself, I'm constantly like, ‘Oh, I forgot how to use this function. Let me just look at the help function’. Almost to give them [students] an insight of like, how I do it, because obviously, I make mistakes when I code. And actually, that causes me a bit of anxiety, that I don’t look like I’m superb, but[…] I feel like it’s actually important for them to see[…]and I do things wrong[…]And then I’ll figure it out” (Teacher9, FoNS).

Through modelling, they highlight how to support students’ cognitive and emotional development in a way that recognises both are interconnected, just as they are for Teacher9. They also demonstrate how teachers balance the risks of such strategies in a culture that so prizes being knowledgeable, with modelling sustainable relationships with exponentially growing, complex STEMM knowledge.

#### Recognising interrelationships between knowing and feeling

Data also gave insight into students’ perceptions of how cognitive and emotional dimensions contribute to achievement of their learning goals and the role that peers play in this process. Table 2 illustrates the ways in which first year medical students, in a module designed for learning how to integrate scientific knowledge to address clinical patient cases, demonstrated varying awareness of the interrelationships between cognition and emotion regulation.

**Table 2:** Illustration of student awareness of interrelationships between cognition and emotion.

Medical student responses to: “How do your student peers contribute to this feeling?”	Learning goals
“We help each other and **fill the gaps** in each other’s knowledge.” “Allows ideas to bounce between so **different perspectives** can be understood.”	Cognition-focussed goals
“They contribute **thoughtfully** and with **sensitive** wording.” “**Safe** to discuss ideas.” “They make me **feel relieved** because they are struggling too.”	Emotion-focussed goals
**“Interesting discussions**, encourages me to **engage** more.” “Some will **explain things** and make me **feel better**.” “We engage in **productive discussion** and **have fun** whilst learning.”	Awareness of interaction between cognition and emotion-focussed goals

Some student responses emphasised the cognitive value that peer interactions held for them (first row) and others’ responses focussed solely on the emotional value of their group’s interaction (second row). The third row shows quotes that illustrate how some student responses recognised the way that cognitive and emotional dimensions interact through the collaborative learning process to support their learning and emotional well-being. We map their responses to learning goals to illustrate that, despite emphasis on knowledge-based learning goals in the official curriculum, students recognise how, as a group, they address their emotional goals and we argue that this provides evidence of valuable emotion socialization.

Within the teachers’ dataset, acknowledgement of emotional support between student peers was absent, yet all teachers demonstrated that working with learner emotion was part of their role. In the quote below, module lead Teacher16 recognises the importance of working with both student and tutor emotion around cognitively demanding integrated knowing. In this way, they facilitate emotion socialization across the whole group.

“we always want [tutors] to relay to the students[…]‘you may ask us a question, we may not know the answer and that’s alright, you know, because no one knows everything. And you need to get used to that as a future doctor’[…]that’s giving a comfort level to your tutors. But I think it’s also[…]managing the emotion of your students” (Teacher16, FoM).

Handled well, as Teacher16, describes, this also creates opportunity to model how clinicians cope with pressure of knowledge overload and uncertainty in professional practice.

#### Knowledge-related emotional connections

A surprise for teachers with whom we shared their students’ data related to students’ perception and understanding of their teachers’ emotional experiences in STEMM classrooms. Student responses gave interesting insight into their teachers’ perceived relationships with knowledge, and cognitive and emotional connection that this created between students and teachers.

“I think my tutor felt energized by the different designs which [our] group is coming up with. My tutor has done research in our area of design interest, so I felt that he relished the chance to reuse old knowledge” (Student187, FoE).

Student perceptions of how their teachers felt, and why, also provided useful information regarding the degree of alignment between their own and teachers’ goals. Student responses again reflected a transition in relation to students’ recognition of knowledge- and cognition-related goals, with some seeing teachers’ goals in terms of them being communicators of knowledge: *“Hopefully [teachers feel] proud of their explanations, as they helped the students (including myself) a great deal to understand the concepts” (Student100, FoNS).*

Whilst other students’ responses, such as Student40’s thoughts regarding their teacher’s emotional experience, suggested that their teacher’s goal was related to students’ construction of solutions, rather than application of defined knowledge, and that students were enabling achievement of this goal: *“Enjoyable as it is open-ended and they get to see students’ solutions”* (Student40, FoE).

### Cognition- and emotion-based comparison

#### Evidence of academic goal achievement

Student data indicated that participants’ tendency to compare their ability and progress towards academic goals with peers contributed significantly to their emotional experiences, both positive and negative, as illustrated earlier by Student117 and Student77.  This is likely to have been heightened in the STEMM learning contexts under focus, where pedagogic methods designed to promote collaborative learning, such as group-based problem-solving, team-based learning and project work were demonstrated as being a double-edged sword. They created opportunities for students to realise what they needed to learn and work on, and provided peers to help with this, but this could feel exposing. The data suggested that students could learn to work with this discomfort collectively, through interacting and shared cognitive and emotional processes, as Student66 implies: *“I think my peers also might have felt overwhelmed during the session however the active discourse between us when answering the problem should have hopefully put them at ease” (Student66, FoNS).*

Our study suggests that in these challenging STEMM learning contexts, social comparison^35^ functioned in complex and contradictory ways and was related to the nature and quality of evidence that students used to judge achievement of their own and others’ goals.

#### Unhelpful social comparison

Student data suggested that they sometimes used the emotions they perceived or inferred in peers as social information to judge their comparative academic ability and progress. Some students also made social comparisons based on assumptions that peers experienced more positive or less negative emotion than them, as Student244 illustrates:

“Good and also a bit miserable. It was a nice seminar. I like when I can talk to people and learn new things from them. But I also felt as if I was doing nothing this whole year as I didn’t remember nearly anything… it was an exciting seminar I wanted to learn more but also the feeling of me being a failure came to the surface…[Peers feel] that they are confident and it’s fun for them” (Student244, FoNS).

Student participants reporting negative emotion were more likely to compare their academic ability unfavorably with that of peers. Despite this, as Student244 demonstrates, negative emotion from cognitive and emotional comparison could be balanced, to some extent, by positive emotion created through social interaction.

In these collaborative learning settings, our data also suggested that many students’ constructions of their emotional experiences were influenced both by comparisons of what they contributed cognitively and emotionally, as well as what they gained. A perceived inability to contribute could result in negative and contradictory emotions, that were not supportive of learning: *“[I feel] Relaxing whilst useless. I'm not making as much contribution like my teammate[…] They’re stronger than me, not that it’s bad, but I just sometime feel like kind of a burden” (Student14, FoE).*

Some responses implied that collaborative learning conferred combined cognitive and emotional loads, that may be under-recognised: *"Highly taxing[…]I have trouble understanding how I contribute" (Student45, FoE).*

Such data also provides more nuanced insight into how control and value influence emotion in learning when students who want to gain and contribute from collaboration feel they cannot.

#### Helpful social comparison

Some students’ responses demonstrated that comparing themselves to peers they perceived as progressing well academically created an opportunity to improve, and these students tended to report cognitions consistent with a growth mindset^36^. Contributing factors included opportunity for repeated practice and tangible evidence of group members’ cognitive development, in Student279’s case, in the form of attempted problem sheets:

“the seminar means easier way to find weakness in learning and solving problems. It is activating, as our peers are pushing me to do better in my study[…]They showed great progress in every week and this helps me to get motivated” (Student279, FoNS).

Student data suggested that social comparison can have more beneficial emotional and cognitive impact when members of the group can make evidence-informed comparisons, as opposed to assuming that peers are ‘naturally better’. Student83’s quote illustrates how some student participants, especially in second year cohorts, recognised that diverse strengths and ways of working could be usefully compared and shared, a process we are calling ‘normalising social comparison’. *“We are all trying and have different strength. Everyone is sharing their thoughts and explaining how they reached each answer” (Student83, FoNS).*

#### Emotion-informed comparison

Many students reported feeling similar to peers and, even when this was not in relation to positive emotions, this sense of emotional connection seemed beneficial. This may have been because others’ emotions informed students’ appraisals of their own ability to cope and achieve their goals^37^, especially with mutual support. They referred to ‘being allies’ and ‘in it together’, suggesting emotional convergence contributed to group identification^38^:

"Everyone feels the same way, so I don’t feel as isolated or concerned that I’m the only one feeling this way. So in that sense there is a group morale in that we all ‘struggle together’ but it’s helpful to lean on others and talk to each other when we’re stressed out” (Student48, FoE).

In some cases, being able to contribute empathetically, in the same way as peers, became a goal: *“[Peers] are non-judgemental and helpful[…]I aim to reflect the same feeling” (Student112, FoNS).*

Teachers did not seem to recognise emotion-informed comparison amongst their students. Teacher1’s example below of a lab-based student group could be interpreted as peers comparing their cognitive understanding and collectively regulating their emotion around fear of looking like they don’t know:

“One of the most annoying things actually, you hear a little group of students telling each other that they don’t know what to do. So "Do you know what to do?" "No, I don’t know what to do." "Do you know what to do?" "No, I don’t know what to do."[…]"Nobody knows what to do." And you’re standing right there and you would help them[…]You go up to them and ask them, "Do you need some help?" Some of them will say ‘yes’, sometimes even still they say ‘no’” (Teacher1, FoNS).

Teacher1’s annoyance, they explained, is because they do not expect students to know and feel that their goal to help is explicit. Although Teacher1, unsurprisingly, did not recognise it, this peer conversation may represent an important example of emotion socialization. Some teachers perceived comparison as competitive behaviour and described feeling frustrated, including because competitiveness was misaligned with teachers’ goals for collaborative learning.

## Discussion and implications for practice

We found, perhaps contrary to popular belief, that being in challenging STEMM contexts were emotional experiences for both teachers and their students, especially when group-based learning involved reciprocal cognitive and emotional interactions. Our data demonstrate that participants in this study experienced diverse and contrasting emotions as they pursued their learning and teaching goals, and that they influenced each other’s emotion through this process. They also demonstrated awareness and understanding of each other’s emotions and even associated goals.

Student responses suggested that they constructed differing emotions in relation to grappling with challenging knowledge and complex cognitive processes. Based on our data, we suggest that students both construct and make meaning of their emotions in relation to their developing understanding of university appropriate, cognitive goals and their changing relationship with knowledge. We hypothesise that this may be partially accounted for by students’ differing and transforming epistemic beliefs. These can range from ‘absolute knowing’, believing knowledge is certain and there is a correct answer, to ‘contextual knowing’, being able to accept uncertainty, and evaluate and apply knowledge within a specific context^39^. It is suggested that these epistemic beliefs and associated goals transform as students progress through university study, influenced by their development and social context^40^. Making this transitional process explicit to students could help them to understand new educational goals, transform epistemic beliefs and regulate associated emotions. Our data demonstrates that peers, with their varied and developing perspectives, are important contributors to this aspect of learning. We suggest that through collaborative learning and resultant emotion socialization, students can develop awareness of how emotion and knowledge-related goals interact and the value of peer support in this. This could be more deliberately communicated through STEMM teaching, including connections to future professional goals. For example, emotional competence is valuable in STEMM-related careers, where ‘contextual knowing’ is often used in combination with emotion to make collaborative decisions and solve problems in the face of inherent uncertainty^41^ and inevitable failed attempts^10^. Teachers should be supported by their universities to model difficulties in working with complex STEMM knowledge, demonstrating ‘intellectual candour’, without feeling extreme discomfort or risking loss of credibility^42^.

Students’ tendency to evaluate progress towards their academic goals through social comparison^43^ led to a range of experiences, some more emotionally and cognitively beneficial than others. Social comparison is especially important at times of uncertainty, when it may be more important to judge whether we are performing at the expected standard^44^. Based on our findings, we recommend offering regular practice opportunities with feedback, and encouraging more evidenced approaches to supplement students’ inferences about peers’ assumed ability and emotions, to foster ‘normalising social comparison’. This included peers discussing how they felt about learning and sharing appropriate strategies. Specifically in group-based learning, conversations about contribution that recognises different strengths and guidance on how to regulate emotions specifically experienced in collaborative learning^3^ is recommended. Teachers should recognise and facilitate helpful social comparison and emotional support between peers. Gathering and sharing anonymised emotion data, such as that generated in this study’s questionnaires, could inform students’ social appraisals of emotion^37^, making them more accurate.

Based on our findings, we suggest that empathy, defined as the capability to perceive, understand and respond to the emotional experiences of others^45^, as our participants variously demonstrated, should be framed as a cognitive competency^46^ that members of STEMM collaborative learning contexts use reciprocally to understand each other’s emotions and associated goals, and collaborate to achieve these. We posit that this could be better supported by more explicit discussion about educational goals in STEMM curricula, including broadening them to promote socioemotional learning. In order to address these goals, equipping students to cope with demands of academic study and professional decision-making, we argue that the highly effective, yet currently invisible, process of emotion socialization should be made more explicit. We have demonstrated that STEMM socializing agents already contribute to this process. Greater acknowledgement of its value would enable all students and teachers to continue developing emotionally and cognitively. The goal here is not uniformity of emotional experience, but to use understanding of emotional variation in learning and teaching as a lens to inform and drive more inclusive practice.

## Limitations

These findings are from a single site study, although we have sampled teaching from multiple STEMM disciplines and teaching contexts, and hope to have offered sufficient insight to enable consideration of the applicability of ideas discussed, and further research, in distinct university contexts. This study also involved purposively selected teachers, whose practice and context were known to us, and who were already using innovative, collaborative approaches, and volunteering students, and we accept that this may have introduced selection bias. Despite this, there was no prior awareness of teachers’ or students’ levels of emotional competence.

## Conclusion

Through our study, we have demonstrated the richness and complexity of connections between cognition and emotion in STEMM university learning and teaching, constructed as individuals continually negotiate relationships with knowledge and evaluate goal achievement, including through comparison. We highlight the benefits, for a group’s cognitive, social and emotional development, of making explicit such connections, that are unlikely to be acknowledged otherwise. This study offers possibilities for capitalising on interactions between university teachers and students and the ways that they reciprocally influence cognition and emotional well-being.

## Supplementary Information

Supplementary Information.

## Data Availability

Data are available from the corresponding author on a reasonable request, within the limits and restrictions of ethical approval.
