# Supplementary material for: Reciprocal cognitive and emotional interaction in STEMM university learning and teaching
Source: Sci Rep. 2024 Sep 17;14:21660. doi: 10.1038/s41598-024-72656-w (PMC11408516; doi:10.1038/s41598-024-72656-w)
Supplement: Supplementary file 1 — Supplementary Information. [file 41598_2024_72656_MOESM1_ESM.pdf]

Reciprocal cognitive and emotional interaction in STEMM university learning and teaching

Kate Ippolito* & Martyn Kingsbury

Imperial College London

*k.ippolito@imperial.ac.uk

Supplementary Information

Data Collection Tools:

Step 1: Teacher Focus Group Interview Schedule

1. How does learning in this module make students feel?

2. How do you know?

3. In what ways do you take these emotions into account when planning / when facilitating / when giving feedback?

4. Is this different in the newer, active learning contexts?

5. How does teaching this module make you feel? Please give a reason for your response. (Give option to speak about how teachers in general feel, rather than describing their own emotions).

6. Do you think that you reveal your emotions in the classroom? If so, when and why? If not, why not? (What conditions are in place?)

Electronic card sort activity using PowerPoint slides (see below).

Activity 1: Identify two emotions that you observe in students and which aspects of the module you think contribute to each emotion (each of the three teacher interviewees respond in turn).

Activity 2: Identify whether you think the emotions on the right have positive or negative valence (make individuals feel pleasant or unpleasant) and whether they have an activating or deactivating effect on learning. (All three participants gave suggestions and one then dragged the emotion to the agreed position on the 2x2 grid. We were as interested in the reasons given for the position of each emotion, as the actual position).

		Emotional Valence	
		**Positive (Pleasant)**	**Negative (Unpleasant)**
**Activation of Learning**	**Activating**		
	**Deactivating**		

Anxiety	Frustration
Boredom	Hope
Confusion	Hopelessness
Curiosity	Pride
Enjoyment	Relief

Activity developed from Pekrun & Perry (2014) and Pekrun et al. (2017).

7. Ideally how do you want students to feel?

8. What can you currently do to try to achieve this? What constrains you? How do you feel about this and why? (if not already raised).

9. What do you think is the relationship between how your learners feel about their learning and their general well-being?

10. What do you think is the role of teaching staff in considering emotion and helping students to regulate their emotions?

11. Anything else you would like to add?

Step 2: Student Questionnaire

1. How has this session made you feel? / How is this project making you feel?

2. Why do you think you feel this way?

3. Do you think this feeling has an activating (makes you want to learn) or deactivating (puts you off learning) effect on your learning? Please give a reason for your response.

4. How does the teacher contribute to this feeling?

5. How do your student peers contribute to this feeling?

6. How do you contribute to this feeling?

7. How do you think your teacher would say they feel whilst teaching this session/project? Please give a reason for your response.

8. How do you think your student peers would say they feel during this session/project? Please give a reason for your response.

9. How does the way this module makes you feel compare with the rest of your degree experience?

Step 3: Follow-up teacher interview

Student questionnaire data and follow-up interview questions shared with teachers before interview.

1. What did you find interesting in the student responses?

2. What did you find surprising?

3. What would you like to do in response to the data, if anything?

Coding Guide:

Themes	Sub-themes	Codes
**Relationships with knowledge**	Developing cognitive goals	Getting lost, not understanding<br><br>Falling behind<br><br>Catching up<br><br>Cognisant of developing understanding<br><br>Building self-efficacy<br><br>Role of peers in knowledge development
	STEMM teachers not knowing everything	Cannot know everything<br><br>Teachers coping with not knowing<br><br>Teachers modelling not knowing<br><br>Teachers making mistakes<br><br>Managing risks in not knowing/making mistakes
	Recognising interrelationships between knowing and feeling	Student's cognition-focussed goals<br><br>Student's emotion-focussed goals<br><br>Teacher's facilitation of goal
	Knowledge-related emotional connections	Student recognises teacher emotion<br><br>Inferring cognitive goals from emotions<br><br>Student attribution for teacher emotion – awareness of knowledge-related goal<br><br>Student attribution for teacher emotion – goal achievement<br><br>Student attribution for teacher emotion – student contribution to goal
**Cognition and emotion-based comparison**	Evidence of academic goal achievement	Knowing how well you're developing cognitively<br><br>Knowing how well you're coping emotionally<br><br>Knowing how peers are developing cognitively<br><br>Knowing how well peers are coping emotionally

	Unhelpful social comparison	Not doing as well as others academically<br><br>Not feeling as positive as peers emotionally<br><br>Not feeling able to contribute cognitively / with knowledge<br><br>Not feeling able to contribute emotionally<br><br>Can't see a way to improve – fixed mindset
	Helpful social comparison	Feeling motivated by peer behaviours and outcomes<br><br>Opportunity to do better<br><br>Understanding how to learn better<br><br>Sharing thoughts, understanding and approaches<br><br>Normalising social comparison
	Emotion-informed comparison	Awareness of how peers feel<br><br>Feeling similar to each other<br><br>Inferring why peers feel the way they do<br><br>Feeling positively influenced by peers<br><br>Comparison of ability to help<br><br>Barriers – Lack of emotional awareness or similarity<br><br>Barriers – Lack of ability to help<br><br>Barriers – Misaligned goals
